# Contextual factors associated with depression among urban refugee and displaced youth in Kampala, Uganda: findings from a cross-sectional study

**DOI:** 10.1186/s13031-020-00289-7

**Published:** 2020-07-10

**Authors:** Carmen H. Logie, Moses Okumu, Simon Mwima, Robert Hakiza, Doreen Chemutai, Peter Kyambadde

**Affiliations:** 1 Factor Inwentash Faculty of Social Work, University of Toronto, 246 Bloor Street West, Toronto, ON M5S 1V4 Canada; 2 Women’s College Research Institute, Women’s College Hospital, 76 Grenville St, Toronto, ON M5G 1N8 Canada; 3 School of Social Work, University of North Carolina, Chapel Hill, 325 Pittsboro St, Chapel Hill, NC 27599-3550 USA; 4 AIDS Control Program, Ministry of Health, Plot 6, Lourdel Road, Nakasero, Kampala, Uganda; 5 Young African Refugees for Integral Development (YARID), Nsambya Gogonya, Kampala, Uganda; 6 Department of Public Administration and Management, Gulu University, Gulu, Uganda; 7 PK is the Executive Director of the Most At Risk Population Initiative (MARPI) Clinic, Mulago Hospital, Kampala, Uganda

**Keywords:** Depression, Youth, Uganda, Refugees, Poverty, Violence, Social support, Food insecurity, Community insecurity, Context

## Abstract

**Background:**

Advancing mental health among refugee and displaced adolescents and youth is critically important, as chronic psychological stress can have lifelong harmful impacts. These groups experience socio-environmental stressors that can harm mental health. Informed by a social contextual framework, this study explored the prevalence of depression among urban refugee and displaced youth in Kampala, Uganda and associations with symbolic (violence), relational (social support), and material (food and community insecurity) contexts.

**Methods:**

We implemented a cross-sectional survey with refugee and displaced adolescent girls and young women and adolescent boys and young men aged 16–24 living in Kampala’s informal settlements. We conducted peer-driven recruitment, whereby peer navigators shared study information with their networks and in turn participants were invited to recruit their peers. We conducted gender disaggregated analyses, including stepwise multiple regression to examine factors associated with depression. We then conducted structural equation modeling (SEM) using weighted least squares estimation to examine direct paths from violence, food insecurity, and community insecurity to depression, and indirect effects through social support.

**Results:**

Among participants (*n* = 445), young women (*n* = 333) reported significantly higher depression symptoms than young men (*n* = 112), including any symptoms (73.9% vs. 49.1%, *p* < 0.0001), mild to moderate symptoms (60.4% vs. 45.5%, *p* = 0.008), and severe symptoms (13.5% vs 3.6%, *p* = 0.002). SEM results among young women indicate that the latent violence factor (lifetime sexual and physical violence) had direct effects on depression and social support, but social support did not mediate the path from violence to depression. The model fit the data well: χ2(3) = 9.82, *p* = 0.020; RMSEA = 0.08, 90% CI [0.03, 0.14], CFI = 0.96). Among young men, SEM findings indicate that food insecurity had direct effects on social support, and an indirect effect on depression through the mediating role of social support. Fit indices suggest good model fit: χ2(3) = 2.09, *p* = 0.352; RMSEA = 0.02, 90% CI [0.000, 0.19], CFI = 0.99.

**Conclusions:**

Findings reveal widespread depression among urban refugee and displaced youth in Kampala, disproportionately impacting young women. Contextual factors, including food insecurity and violence, increase depression risks. Strategies that reduce gender-based violence and food insecurity, and increase social support networks, have the potential to promote mental health among urban refugee and displaced youth.

## Background

A constellation of factors contribute to psychological distress among refugee and displaced youth—including displacement, trauma, violence, community insecurity, and healthcare barriers [1]. Halting psychological distress and promoting mental wellbeing among refugee and displaced adolescents and youth is critically important, as chronic psychological stress in this developmental phase can have harmful and lifelong impacts on neurobiological systems connected with emotional and behavioural regulation [2]. Yet most youth in humanitarian settings [3]–and non-conflict settings [4, 5]—do not receive needed mental health support. As mental health issues are the foremost cause of youth disability across global regions—comprising 45% of the overall disease burden [6]—increased knowledge of intervenable factors to promote refugee and displaced youth mental health is a global health priority.

Uganda is an important case study to understand refugee and displaced youth mental health, as it is the third-largest refugee hosting nation in the world, hosting an estimated 1.39 million refugees at the end of January 2020 [7]. Advancing refugee health is a national priority in Uganda, with the goal that refugees will benefit from social services and access to durable solutions [8]. Globally there is a phenomenon of refugee and displaced urbanization—60% of the world’s refugees and 80% of internally displaced persons live in urban settings [9]. In Uganda, the urban city of Kampala hosts 78,501 refugee and displaced persons; this includes both refugees (*n* = 59,923) and people seeking asylum (*n* = 18,578) [7]. More than one-quarter (28%) of Kampala’s urban refugee and displaced persons are youth aged 15–24 [7]. Most refugee and displaced persons in Kampala live in informal settlements, also known as ‘slums’ [10]. Social and environmental stressors in slums—such as elevated exposure to violence and poverty—may contribute to mental health challenges and yet are underexplored with urban refugee and displaced youth [11].

The United Nations defines a slum as “a contiguous settlement where the inhabitants are characterised as having inadequate housing and basic services.” [12] The term slum itself is contested and may be considered stigmatizing, leading to suggestions to use the term ‘informal settlement’ [11]. Yet researchers [11] and the United Nations continue to refer to slum health [13]. Over half (56%) of Sub-Saharan Africa’s urban population lives in slums [14] that are often characterized by overcrowding and limited sanitation, constrained healthcare access, higher rates of violence, and limited formal employment opportunities that result in precarious employment such as hawking, washing clothes, and survival sex [11]. Neighbourhood effects in slums refer to the ways that community-level health is affected by closely shared social and physical environments, independently from individual household factors. Due to high population density, neighbourhood effects can result in the spreading of illness and poor physical health outcomes (e.g., malnutrition, diarrhea) [15]. Overcrowding in slums can also lead to stress, which in turn can contribute to mental health challenges [11]. There are calls for research specifically focused on slums as “spatial entities” with unique needs and lived realities in comparison with non-slum urban areas [15].

Mental health in slums is understudied at large [11, 16], and this is also true in Uganda among both refugee/displaced and non-refugee/displaced persons. Studies of health issues, including mental health, among refugees and displaced persons in Uganda have focused mainly on persons living in refugee settlements [17]. For instance, in a study with refugee adolescents in Northern Ugandan settlements, one-quarter reported high rates of emotional symptoms and stress and one-fifth anxiety—exposure to violence was associated with both stress and anxiety [18]. Qualitative research with urban refugees in another context, Nairobi, reported that community insecurity and violence were collective challenges that harmed mental health [19]. Although safety concerns are reported in some slums, there is heterogeneity within the social and physical environments of slums that underscores the importance of considering context [15]. Safety concerns in non-slum, non-refugee/displaced settings have been associated with depressive symptoms [20], and this may, in part, be due to social isolation and mistrust. Another issue facing people living in slums in diverse locations such as India [21], Kenya [22], and Uganda [23, 24] is food insecurity. In turn, food insecurity is associated with elevated vulnerability to mental health challenges [25]. A study with adolescent girls living in slums in India reported associations between food insecurity and depression [26]. There is an urgent need to understand intervenable contextual factors that shape mental health with urban refugee and displaced youth living in slums to inform tailored programming.

This study is informed by a social contextual approach conceptualized by Campbell and Cornish [27]. In ‘receptive social environments’ persons have increased agency over their health and wellbeing [27, 28]. Such supportive and health-enabling social contexts include equitable social relationships. Three elements of social context emerge as important in understanding health promotion [27, 29]. *Material contexts* include access to resources, such as money, food, housing, and other economic opportunities. *Relational contexts* include social capital and social relationships, including with friends, families, and external actors. Finally, the *symbolic context* refers to larger cultural worldviews and ideologies, including gender equity and recognition of one’s worth, value, dignity, and rights. This approach may be particularly important for conceptualizing mental health among refugee and displaced youth. For instance, symbolic contexts that reduce the rights and dignity of refugee and displaced youth and contribute to depression include experiences of violence [18, 19]. Food insecurity [25] and community insecurity [19, 20] reflect material contexts associated with depression. Finally, relational contexts such as social support may be a protective factor for depression [30–32], including in stressful neighbourhoods [33].

To our knowledge, pathways between symbolic, material, and relational contextual factors and depression are understudied among urban refugee and displaced youth in Uganda. There are also knowledge gaps regarding gender differences in prevalence and correlates of urban refugee and displaced youth’s mental health. To address these knowledge gaps, we explored a) prevalence of depression among urban refugee and displaced youth, b) associations between symbolic contexts (childhood abuse, sexual violence, physical violence), relational contexts (social support), and material contexts (food insecurity, community insecurity) with depression; and c) direct paths from violence, food insecurity and community insecurity to depression, and indirect paths through the mediating role of social support.

## Methods

### Participants

We undertook a community-based survey between January and March 2018 with refugee and displaced youth aged 16–24 living in five informal settlements in Kampala, where most refugee/displaced persons in the city reside. Community collaborating partners included refugee-focused agencies (Interaid Uganda, Young Africans for Integral Development [YARID], Tomorrow Vijana), and government agencies (Uganda AIDS Control Program, Ministry of Health). We surveyed youth who self-identified as: aged 16–24; a refugee or displaced person or having refugee/displaced parents; living in one of 5 informal settlements in Kampala (Kabalagala, Rubaga, Kansanga, Katwe or Nsambya); and able to provide informed consent.

### Recruitment

We collaborated with community partners for recruitment and survey implementation. We trained 12 peer researchers who lived in the five target informal settlements (four young men and eight young women) who identified as a refugee or displaced person aged 18–24.

Data are from the Maono Ya Pamoja (A Shared Vision) study focused on HIV among refugee/displaced youth in Kampala. As AGYW are twice as likely to report new HIV new infections compared with ABYM in Uganda [34], we intentionally oversampled for proportional representation of AGYW. Our training with peer researchers included research methods, ethics, and data collection. Recruitment followed peer network sampling [35] strategies, an approach whereby peers with shared life experiences (in this study, refugees) use word of mouth for recruitment, and newly recruited participants are invited to share the study with their own peers by word of mouth. Peer driven approaches are an effective strategy for recruiting and including marginalized populations where there is no sampling frame, such as with urban refugee and displaced youth in Kampala. In this study, peer researchers and community partners shared the study information with their social networks, and each participant received 2–5 study ‘coupons’, designed to look like movie tickets, to help with recruiting their peers for study participation. Coupons shared the peer researcher contact details. We received research ethics approval from the University of Toronto and the Ugandan Ministry of Health.

### Data collection procedures

The peer research assistants administered android tablet-based surveys using the Quicktaps platform to participants at various community-based locations (e.g., community agencies), selected by the participant. Surveys were 35–40 min and conducted in English, French, or Swahili. Informed consent was provided on the tablet-based survey before administration, and participants received information about psychosocial resources, including referrals for mental health first aid. The research coordinator, a trained social worker, was available to provide any needed support in times of distress. There were no reported distress incidences during the administering of the survey. Participants received an honorarium of UGX 12,500-shillings (∼USD 3.72), an amount recommended by community partners.

### Measures

The **outcome variable** was *depression symptoms* assessed using the Patient Health Questionnaire-9 (PHQ-9) [36]. Following scoring guidelines, participants were categorized with the following symptoms: minimal symptoms (no depression diagnosis) (1–4), mild to moderate depression (5–14), and moderately severe to severe depression (15–27) (Cronbach *α* = 0.87).

Informed by the guiding social contextual theoretical framwork [27], the **explanatory variables of interest** included material (food insecurity, community insecurity) and symbolic (childhood sexual abuse, lifetime sexual violence, lifetime physical violence, lifetime verbal abuse) contextual factors.

*Food insecurity* was assessed with a single item asking participants how often they went to sleep hungry because they did not have enough food to eat (responses were dichotomized for those who indicated they had gone to sleep hungry rarely- always [1] or never [0]). Food insecurity is an indicator of poverty assessed using this single item in prior research [37, 38]. *Community insecurity* was assessed by asking participants to indicate how physically safe they felt in their communities (responses were dichotomized: not safe = 1, and fairly safe-very safe = 0).

We assessed *experiences of violence* (physical, verbal, and/or sexual) at the age of 16 years and over. Participants were asked: *When you were 16 or older, have you ever experienced (check all); sexual violence (yes = 1, no = 0); physical violence (yes = 1, no = 0); or verbal abuse (yes = 1, no = 0). Experiencing childhood sexual abuse* was assessed by asking participants if they had experienced sexual abuse before the age of 16 *(yes = 1, no = 0)*.

The **mediating role** of the *relational context* was assessed with *social support*, measured using the 24-item multidimensional scale of perceived social support (MSPSS) that measures support from three sources (family, friends, and a significant other) [39]. In this study, the social support scale had high reliability (Cronbach *α* = 0.91), with higher scores indicating greater social support. Subscales also had high reliability (family: Cronbach *α* = 0.85, friends: Cronbach *α* = 0.85, and significant other: Cronbach *α* = 0.88).

*Sociodemographic* variables included age (continuous), an education level (categorical: no education/less than secondary school, and post-secondary education) and time in Uganda (less than 1 year, 1–5 years and more than 5 years).

### Data analysis

We conducted gender disaggregated analyses [40] to understand shared and different contextual factors associated with depression. We first conducted bivariate analyses to identify differences in depression scores using Stata 14.0. We used variance inflation factors (VIFs) to check the multicollinearity of the explanatory variables. The VIF results indicated no concerning correlations (VIFs< 1.6). Subsequently we conducted stepwise multiple regression analyses stratified by gender to identify factors associated with depression.

Following this we conducted structural equation modelling (SEM), using MPLUS 8, with the means and variance adjusted weighted least squares (WLSMV) estimation method due to nonnormal data [41]. In SEM we examined direct paths from violence, food insecurity, and community insecurity to depression, and indirect effects through social support. Each model only included variables significantly associated with depression in the multiple regression analyses. For SEM models that included variables reflecting the same underlying factor, we developed latent variables. Using latent variables in SEM can reduce measurement error, address collinearity problems with multiple indicators, and examine the synergistic effects of multiple indicators [41]. Building on multiple regression findings, we developed a latent construct of violence for the AGYW analysis (indicators: sexual violence, physical violence) and for the ABYM analysis a latent construct of social support (indicators: family support, significant other support).

For SEM, we used three fit indices to evaluate the fit of the measurement and structural model: root mean square error of approximation (RMSEA) (acceptable if 0.08 to 0.10, good if ≤0.05), comparative fit index (CFI) (acceptable if >0.90, good if > 0.95) [42]. Although Chi square is often used as a measure of fit, due to its sensitivity to sample size it is not recommended when the sample size is larger than 200, as it is generally significant (such as the current sample size for AGYW) [43]. For all analyses, statistical significance was set at the *p* <0.05 level.

## Results

### Study population

Table 1 reports sociodemographic characteristics and contextual factors for the entire sample (*N* = 445) and bivariate analysis results of differences by gender. Participants included adolescent girls and young women (AGYW) (*n* = 333; 74.8%) and adolescent boys and young men (ABYM) (*n* = 112; 25.2%). Two-thirds of all participants reported depression symptoms: over half reported mild to moderate depression symptoms (56.6%), 11.0% severe depression symptoms, and 32.4% reported no depression symptoms. AGYW reported significantly more depression symptoms than ABYM, including: any depression symptoms (73.9% vs. 49.1%, *p* < 0.0001), mild to moderate depression symptoms (60.4% vs. 45.5%, *p* = 0.008), and severe depression symptoms (13.5% vs 3.6%, *p* = 0.002). In bivariate analyses, factors associated with depression among AGYW included: age; lifetime sexual, verbal and physical violence; childhood verbal abuse; community insecurity; and friends, significant other, and family social support dimensions. For ABYM, in bivariate analyses depression was associated with: childhood sexual abuse; food insecurity; and significant other and family social support dimensions.

**Table 1:** Overview of factors associated with depressive symptoms among urban refugee and displaced youth in Kampala, Uganda (*N* = 445)

	Adolescent Boys & Young Men (***n*** = 112)				Adolescent Girls & Young Women (***n*** = 333)			
Indicators	No depression symptoms (*n* = 57; 50.9%) N (%)/ Mean (SD)	Mild to moderate depression symptoms (*n* = 51; 45.5%) N (%)/ Mean (SD)	Severe depressive symptoms (n = 4; 3.6%) N (%)/ Mean (SD, range)	*P* value	No depression symptoms (*n* = 87; 26.1%) N (%)/ Mean (SD)	Mild to moderate depression symptoms (*n* = 201; 60.4%) N (%)/ Mean (SD)	Severe depressive symptoms (*n* = 45; 13.5%) N (%)/ Mean (SD)	*P* value
Age	20.12 (2.31)	20.67 (2.66)	22.25 (2.87)	.187	18.67 (2.33)	19.62 (2.58)	19.16	.014
Education				.414				.526
*> secondary school*	19 (33.3)	23 (45.1)	2 (50.0)		50 (57.5)	111 (55.2)	29 (64.4)	
*Secondary school*	38 (66.7)	28 (54.9)	2 (50.0)		37 (42.5)	90 (44.8)	16 (35.6)	
Time in Uganda				.773				.068
< 1 year	7 (12.3)	4 (7.8)	1 (25.0)		10 (11.5)	10 (5.0)	3 (6.7)	
1–5 years	39 (68.4)	38 (74.5)	2 (50.0)		46 (52.9)	121 (60.2)	19 (42.2)	
> 5 years	11 (19.3)	9 (17.6)	1 (25.0)		31 (35.6)	70 (34.8)	23 (51.1)	
Employment				.425				.062
Unemployed	20 (40.0)	22 (53.7)	2 (50.0)		28 (32.2)	80 (39.8)	24 (53.3)	
Employed/Student	30 (60.0)	19 (46.3)	2 (50.0)		59 (67.8)	121 (60.2)	21 (46.7)	
Lifetime sexual violence				.858				.000
No	54 (94.7)	49 (96.1)	4 (100.0)		84 (96.9)	180 (89.6)	28 (62.2)	
Yes	3 (5.3)	2 (3.9)	0 (0.0)		3 (3.4)	21 (10.4)	17 (37.8)	
Lifetime verbal abuse				.140				.019
No	29 (50.6)	35 (68.6)	3 (75.0)		58 (66.7)	98 (48.8)	25 (55.6)	
Yes	28 (49.1)	16 (31.4)	1 (25.0)		29 (33.3)	103 (51.2)	20 (44.4)	
Lifetime physical violence				.951				.000
No	40 (70.2)	37 (72.5)	3 (75.0)		80 (92.0)	177 (88.1)	26 (57.8)	
Yes	17 (29.8)	14 (27.5)	1 (25.0)		7 (8.0)	24 (11.9)	19 (42.2)	
Childhood sexual abuse				.044				.880
No	57 (100.0)	46 (90.2)	4 (100.0)		73 (83.9)	173 (86.1)	38 (84.4)	
Yes	0 (0.0)	5 (9.8)	0 (0.0)		14 (16.1)	28 (13.9)	7 (15.6)	
Childhood physical abuse				.275				.728
No	34 (59.6)	25 (49.0)	1 (25.0)		39 (44.8)	99 (49.3)	23 (51.1)	
Yes	23 (40.4)	26 (51.0)	3 (75.0)		48 (55.2)	102 (50.7)	22 (48.9)	
Childhood verbal abuse				.853				.044
No	35 (61.4)	31 (60.8)	3 (75.0)		36 (41.4)	89 (42.8)	28 (62.2)	
Yes	22 (38.6)	20 (39.2)	1 (25.0)		51 (58.6)	115 (57.2)	17 (37.8)	
Food insecurity				.037				.208
No	21 (36.8)	9 (17.6)	0 (0.0)		31 (35.6)	51 (25.4)	13 (28.9)	
Yes	36 (63.2)	42 (82.4)	4 (100)		56 (64.4)	150 (74.6)	32 (71.1)	
Community insecurity				.903				.003
No	47 (82.5)	40 (80.0)	3 (75.0)		63 (72.4)	106 (52.7)	21 (46.7)	
Yes	10 (17.5)	10 (20.0)	1 (25.0)		24 (27.6)	95 (47.3)	24 (53.3)	
Social Support								
*Friends*	11.35 (1.72)	10.84 (1.49)	11.25 (1.50)	.263	11.18 (1.66)	10.78 (1.99)	9.36 (3.02)	.000
*Significant Other*	11.80 (1.38)	11.09 (2.07)	9.25 (3.40)	.008	11.09 (1.89)	10.96 (2.70)	9.13 (3.58)	.017
*Family*	12.17 (1.63)	10.94 (1.88)	9.25 (2.22)	.000	11.62 (1.52)	11.58 (2.64)	10.40 (3.74)	.000

*p < .05, **p < .01, ***p < .001

### Multiple regression results of factors associated with depression among refugee/displaced adolescent girls and young women 

Table 2 presents stepwise multiple regression analysis findings regarding factors associated with depression among AGYW. In model 1, lifetime sexual violence and physical violence (*symbolic context*) were associated with depression. After adding community insecurity and food insecurity (*material context*) in the model, lifetime sexual and physical violence remained statistically significant; however, there was an observed reduction in the beta coefficient of lifetime sexual violence from 0.23 to 0.22. Also, community insecurity was associated with increased depression. In model 3, when social support dimensions (*relational context*) were added to the model, there were observed reduction in the beta coefficients for lifetime sexual and physical violence from 0.22 to 0.19. We also observed a reduction in the beta coefficient for community insecurity from 0.12 to 0.11. The friends’ social support dimension was significantly associated with reduced depression. On average, a one-point increase in social support (friends) was associated with a 0.14 decrease in depression, holding other factors constant (β = − 0.14, *p* < 0.050). There was an observed increase in the Adjusted R^2^ from 0.15 (model 1) to 0.18 (model 3), suggesting the final model explained more variance in depression.

**Table 2:** Multivariate regression analysis of contextual factors associated with depression among urban refugee and displaced adolescent girls and young women in Kampala (n = 333)

Indicators	Model 1 ***Beta (SE)***	Model 2 ***Beta (SE)***	Model 3 ***Beta (SE)***
Age	.04 (.122)	.05 (.12)	.04 (.12)
Childhood verbal abuse	−.08 (.70)	−.07 (.07)	−.05 (.70)
Lifetime sexual violence	.23 (.98)***	.22 (.99)***	.19 (.99)***
Lifetime verbal abuse	.08 (.69)	.08 (.69)	.08 (.68)
Lifetime physical violence	.22 (.92)***	.22 (.92)***	.19 (.93)***
Community insecurity		.12 (.64)*	.11 (.63)*
Food insecurity		−.02 (.69)	−.06 (.72)
Friends’ social support			−.14 (.17)*
Family social support			−.06 (.14)
Intercept	5.37 (2.41)*	4.70 (2.46)	11.21 (3.24)***
R-squared	.16	.17	.20
R-squared (Adj.)	.15	.16	.18
N	333	333	333

*p < .05, **p < .01, ***p < .001; All models use standardized beta coefficient

### Multiple regression results of factors associated with depression among refugee/displaced adolescent boys and young men 

Table 3 presents stepwise multiple regression findings of factors associated with depression among ABYM. In Model 1, childhood sexual abuse was associated with increased depression, controlling for age (β = 0.20, *p* < 0.050). In Model 2, after adding food and community insecurity to the model, childhood sexual abuse remained significantly associated with depression, with a reduced beta coefficient from 0.20 to 0.19. Also, the relationship between age and depression became statistically significant (β = 0.19, *p* < 0.050). Food insecurity was associated with increased depression scores 0.24 points higher than non-food insecure participants, adjusting for other variables (β = 0.24, *p* < 0.010). In Model 3, when social support dimensions (significant other, family) were added, age, childhood sexual abuse, and food insecurity became non-significant. Instead, in adjusted analyses, social support (significant other dimension) (β = − 0.22, *p* < 0.050) and social support (family dimension) (β = − 0.22, *p* < 0.050) were associated with reduced depression. Adjusting for other variables, a 1-point increase in either significant other or family social support dimensions was associated with a 0.22-point decrease in depression. The final model (Model 3) explained greater variance in depression (Adjusted R square = 0.24) than the other models (Model 1: 0.06, Model 2: 0.12).

**Table 3:** Multivariate regression analysis of contextual factors associated with depression among urban refugee and displaced adolescent boys and young men in Kampala (*n* = 112)

Indicators	Model 1 ***Beta (SE)***	Model 2 ***Beta (SE)***	Model 3 ***Beta (SE)***
Age	.17 (.168)	.19 (.17)*	.16 (.16)
Childhood sexual abuse	.20 (2.03)*	.19 (1.97)*	.15 (1.85)
Community insecurity		.10 (1.05)	.11 (.98)
Food insecurity		.24 (.93)**	.15 (.88)
Significant other social support			−.22 (.23)*
Family social support			−.22 (.23)*
Intercept	−.51 (3.45)	−3.43 (3.50)	10.31 (4.65)*
R-squared	.07	.15	.28
R-squared (Adj.)	.06	.12	.24
N	112	111	111

*p < .05, **p < .01, ***p < .001; All models use standardized beta coefficient

### Structural equation model on depression among refugee adolescent girls and young women in Kampala with a latent construct of violence

**Fig. 1:**
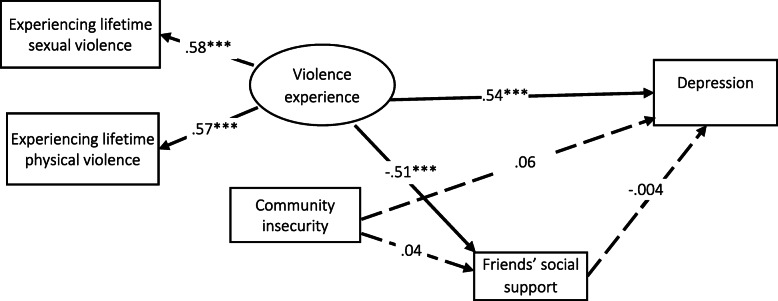
Final path analysis of the relationship between a latent construct of violence, community insecurity, depression, and the proposed mediator of social support among urban refugee and displaced adolescent girls and young women in Kampala, Uganda (*N* = 333)

We conducted SEM with AGYW to determine the direct and indirect effects of a latent construct of violence (indicators: sexual violence, physical violence) and community insecurity on depression, and indirect pathways through the mediating role of friends’ social support. Analyses were adjusted for age. The model fit the data well: χ^2^(3) = 9.82, *p* = 0.020; RMSEA = 0.08, 90% CI [0.03, 0.14], CFI = 0.96. Table 4 presents these SEM findings. There was a direct relationship between violence and increased depression (β = 0.54, *p* < 0.0001) and a direct pathway from violence to lower social support (friends) (β = − 0.51, p < 0.0001). Social support (friends) was not associated with depression (β = − 0.004, *p* = 0.961), thus did not mediate the relationship between violence and depression. Community insecurity was not directly associated with depression (β = 0.06, *p* = 0.296) or social support (friends) (β = − 0.04, *p* = 0.453). The final SEM path tested is illustrated in Fig. 1.

**Table 4:** Final path model parameter estimates of the relationship between a latent construct of violence, community insecurity, depression, and the proposed mediator of friends’ social support among urban refugee and displaced adolescent girls and young women in Kampala, Uganda (*N* = 333)

Paths	β (Standard Error)	Critical ratio	***P*** value
**Direct effect**			
Violence → friends’ social support	−0.51 (0.07)	−7.04	**0.000**
Community insecurity→ friends’ social support	−0.04 (0.06)	−0.75	0.453
Violence → depression	0.54 (0.09)	5.49	**0.000**
Friends’ social support → depression	−0.004 (0.08)	− 0.05	0.961
Community insecurity→ depression	0.06 (0.06)	1.04	0.296
**Indirect effect**			
Violence → friends’ social support → depression	0.002 (0.04)	0.05	0.961
Community insecurity → friends’ social support → depression	0.00 (0.004)	0.05	0.962

### Structural equation model on depression among refugee adolescent boys and young men in Kampala with a latent construct of social support

**Fig. 2:**
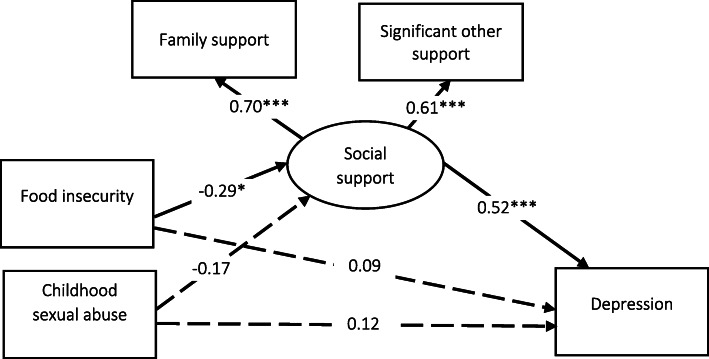
Final path analysis of the relationship between food insecurity, childhood sexual abuse, depression, and the proposed mediator of a latent construct of social support among urban refugee and displaced adolescent boys and young men in Kampala, Uganda (N=111). Note: *p<.05, **p<.01, ***p<.001

We conducted SEM with ABYM to examine the direct and indirect effects of childhood sexual abuse and food insecurity on depression, and the mediating role of the latent social support (indicators: family support, significant other social support). The model fit the data well: χ2(3) = 2.09, *p* = 0.352; RMSEA = 0.02, 90% CI [0.000, 0.19], CFI = 0.99. SEM findings are reported in Table 5. Food insecurity was associated with lower social support (β = − 0.29, *p* < 0.050), but not with depression (β = 0.09, *p* = 0.358). Social support was associated with lower depression (β = − 0.52, *p* < 0.001) and mediated the pathway from food insecurity to depression (Sobel test: β = 0.15, *p* = 0.044). Childhood sexual abuse was not associated with social support (β = − 0.17, *p* = 0.143) or depression (β = 0.12, *p* = 0.176). Figure 2 illustrates the final SEM path tested.

**Table 5:** Final path model parameter estimates of the relationship between food insecurity, childhood sexual abuse, depression, and the proposed mediator of a latent construct of social support among urban refugee and displaced adolescent boys and young men in Kampala, Uganda

Paths	β (Standard Error)	Critical ratio	***P*** value
**Direct effect**			
Food insecurity → social support	-0.64 (0.26)	-2.45	**0.014**
Childhood sexual abuse → social support	-0.83 (0.57)	-1.47	0.141
Food insecurity → depression	0.20 (0.22)	0.92	0.357
Childhood sexual abuse → depression	0.59 (0.43)	1.36	0.174
Social support → depression	-0.52 (0.11)	-4.69	**0.000**
**Indirect effect**			
Food insecurity → social support → depression	0.15 (0.07)	2.01	**0.044**
Childhood sexual abuse → social support → depression	0.09 (0.06)	1.38	.167

## Discussion

Findings reveal widespread depression symptoms among urban refugee and displaced youth in Kampala associated with violence and food insecurity. These contextual stressors include both cumulative (histories of violence) and daily (food insecurity) factors that reveal an accumulation of risk exposures among urban refugee/displaced youth that elevate depression risks. These stressors and depression symptoms require urgent attention, as chronic psychological stressors can harm neurobiological systems linked with emotional and behavioural regulation and their dysregulation is associated with developing psychopathologies [2]. Notably our study applied gender-stratified analyses [40] that revealed important differences in prevalence and correlates of depression symptoms with urban refugee and displaced youth. These findings can inform research and practice to advance mental wellbeing among urban refugee and displaced youth at large, with a particular relevance for Kampala. Due to the shared social and physical environments of slums, findings can also inform interventions to advance adolescent and youth mental wellbeing in slums/informal settlements [15].

Our findings indicate that urban refugee and displaced AGYW were 1.5 times more likely to report depressive symptoms than their ABYM counterparts. A similar trend was found in a Northern Ugandan settlement whereby women had 1.7 fold the odds of reporting major depression than men [44]. The finding that three-quarters of AGYW in our study reported depression symptoms is alarming and calls for urgent attention. While this proportion is higher than the 29% reported among adult women in Northern Uganda [44, 45], it is also difficult to make direct comparisons as we included persons with major depression disorders scored as mild, moderately severe and severe, while it is not clear of the severity levels of major depression in the Northern Ugandan study. The reasons behind women’s global experiences of a higher prevalence of depression than men are contested, some situate these differences within social contexts, psychological factors, sociocultural roles and expectations, and inequitable power dynamics and labour distribution [1, 46]. Our findings reveal contextual factors uniquely associated with depression among AGYW and ABYM.

For instance, our finding that violence was associated with depression among AGYW aligns with the rich evidence base about the sequalae of experiencing SGBV in adolescence and young adulthood, including risk of long-term health challenges such as depression [44]. This finding also expands knowledge of violence and associated mental health challenges in rural refugee settlements [18] to consider analogous experiences of SGBV in slums/informal settlements [15]. This finding corroborates qualitative research by Im et al. [19] with urban refugees in Nairobi that discuss SGBV, lack of support, and community level insecurities that harm mental wellbeing. This Nairobi-based study identified cumulative (war stressors) and current (daily community violence) stressors that disproportionately targeted urban refugee girls and young women [19]. Qualitative research with urban refugee youth in Kampala is needed to further explore how histories of violence are experienced and influence mental health, and to elicit youth perspectives on community solutions.

Among boys we found that food insecurity was associated with depression through the pathway of reduced social support. This corroborates global research that has consistently documented complex linkages between food insecurity and depression [45, 47]. Research has identified bidirectional relationships between depression and food insecurity, whereby food insecurity is a toxic, extreme and chronic stressor that contributes to anxiety, depression and suicidality [45, 47]. Food insecurity can also contribute to shame, powerlessness, and can exacerbate tensions and disparities within families [45]. Depression and mental health challenges can also reduce capabilities to obtain and maintain regular employment, in turn elevating risks of food insecurity. Most food insecurity studies have focused on women [45, 47], yet a global review did not find sex differences in the associations between food insecurity and mental health. Future research can explore the gendered dynamics [40] between food insecurity, reduced social support, and depression among urban refugee and displaced youth, and why food insecurity has particularly detrimental impacts on ABYM’s mental wellbeing. Overall findings signal an urgent need to address food insecurity among urban refugee and displaced youth—75% of ABYM and 71% of AGYW reported food insecurity.

Finally, social support holds promise as an intervenable factor that could mitigate the effects of harmful contextual factors on wellbeing. Lifetime violence experiences among AGYW were associated with lower social support from friends. It is plausible that SGBV stigma and gender inequitable social norms may increase shame and social isolation among AGYW [46], creating barriers to accessing social support from peers. Food insecurity can also lead to blame, guilt and shame, in turn contributing to social isolation [47]. This may explain the finding that food insecurity was associated with lower social support among ABYM. Social support mediated the pathway from food insecurity to depression, signalling its role as a protective factor for boys’ mental wellbeing. Taken together these findings underscore the importance of relational contextual factors in shaping mental wellbeing among urban refugee and displaced youth.

Programs to advance urban refugee and displaced mental health can: a) be gender-tailored, examining the unique experiences of young men and young women, including stressors and coping strategies; b) provide both mental health support and entrepreneurship training and financial resources for economic security; c) provide group-based programs for youth to build social support networks; d) offer family support groups and family resources to support youth mental health. Building sustainable social support networks and healthy family dynamics among urban refugee and displaced youth—and other youth—in informal settlements in Kampala could result in-far reaching benefits beyond mental health. For instance, grassroots networking and organizing has contributed to rights improvement for women labourers in Nairobi slums and sex worker protections in slums in Zimbabwe [12].

There are several study limitations. Due to our non-random sampling approach our study results cannot be extrapolated to all urban refugee/displaced youth. Respondent driven sampling approaches, compared with peer driven sampling, could have helped with controlling for differences due to network size and group clustering [35]. Due to the cross-sectional design we cannot ascertain causality, and there may be bi-directional associations between variables that we were unable to measure (for example, between food insecurity and depression). We only assessed depression, and a fuller picture of mental health could be acquired if we had also measured anxiety and post-traumatic stress disorder. Our brief food insecurity measure, while used in prior studies [37, 38], does not provide information about the scope, persistence, and nature of food insecurity. Similarly, our single-item measure of perceived community insecurity could be expanded in future research to better understand community safety concerns. The violence measures were also single-item, and may have overlooked variations in the types, extent, levels and perpetrators of violence. We pilot tested the survey and made the decision with community partners to shorten the survey to increase feasibility and reduce participant burden. This aligns with research that demonstrates how shorter surveys can improve response and completion rates [48]. Multi-dimensional, standardized measures could be used in future research. Future studies can employ longitudinal approaches to better understand changing dynamics of depression and to assess causal pathways.

During the COVID-19 pandemic, social control policies such as physical distancing and lockdowns may negatively impact mental health [49]. With Uganda imposing an extended national lockdown, studies are needed to address pandemic stress, economic insecurity, and violence experiences associated with lockdown policies during COVID-19. Despite these limitations, our survey expands the knowledge base on contextual factors associated with depression among urban refugee and displaced youth, and underlines the importance of applying a gender-based analysis [40] to refugee/displaced youth mental health.

## Conclusions

Contextual factors, including food insecurity and violence, increase depression risk among urban refugee and displaced youth. While universal challenges associated with displacement to urban contexts may include living in slums and their associated stressors, it is important to explore the unique social and physical environments of slums/informal settlements as these contexts vary in perceived safety, social cohesion, and related health outcomes [12]. Gender differences we noted across variables, particularly regarding AGYW’s higher depression, signal important differences within the same context. This underscores the importance of developing contextually, age and gender specific mental wellbeing interventions in partnership with urban refugee youth. There is a pressing need for research to examine slum/informal settlement environments and pathways to mental wellbeing. The shared social and physical environments in slums/informal settlements allow for diffusion of interventions across neighbourhoods, contributing to economies of scale whereby refugee and non-refugee persons would benefit [15]. Attending to contextual factors through reducing food insecurity and SGBV, and building social support networks, has the potential to advance mental wellbeing among urban refugee and displaced adolescents and youth.

## Data Availability

The datasets generated and/or analysed during the current study are not publicly available due to research ethics board restrictions but are available from the corresponding author on reasonable request and on attaining research ethics board amendments from the University of Toronto and Uganda Ministry of Health.
